# PARP Inhibitors Talazoparib and Niraparib Sensitize Melanoma Cells to Ionizing Radiation

**DOI:** 10.3390/genes12060849

**Published:** 2021-05-31

**Authors:** Stephanie Jonuscheit, Tina Jost, Fritzi Gajdošová, Maximilian Wrobel, Markus Hecht, Rainer Fietkau, Luitpold Distel

**Affiliations:** Department of Radiation Oncology, University Hospital Erlangen, Friedrich-Alexander-Universität Erlangen-Nürnberg, 91054 Erlangen, Germany; Stephanie.jonuscheit@gmx.de (S.J.); tina.jost@uk-erlangen.de (T.J.); fritzizorn@gmx.de (F.G.); Maximilian.Wrobel@stud.uni-regensburg.de (M.W.); markus.hecht@uk-erlangen.de (M.H.); rainer.fietkau@uk-erlangen.de (R.F.)

**Keywords:** PARP, kinase inhibitors, ionizing radiation

## Abstract

(1) Background: Niraparib and Talazoparib are poly (ADP-ribose) polymerase (PARP) 1/2 inhibitors. It is assumed that combining PARP inhibitors with radiotherapy could be beneficial for cancer treatment. In this study, melanoma cells were treated with Niraparib and Talazoparib in combination with ionizing radiation (IR). (2) Methods: The effects of Talazoparib and Niraparib in combination with IR on cell death, clonogenicity and cell cycle arrest were studied in healthy primary fibroblasts and primary melanoma cells. (3) Results: The melanoma cells had a higher PARP1 and PARP2 content than the healthy fibroblasts, and further increased their PARP2 content after the combination therapy. PARP inhibitors both sensitized fibroblasts and melanoma cells to IR. A clear supra-additive effect of KI+IR treatment was detected in two melanoma cell lines analyzing the surviving fraction. The cell death rate increased in the healthy fibroblasts, but to a larger extent in melanoma cells after combined treatment. Finally, a lower percentage of cells in the radiosensitive G2/M phase is present in the healthy fibroblasts compared to the melanoma cells. (4) Conclusions: Both PARP inhibitors sensitize melanoma cells to IR. Healthy tissue seems to be less affected than melanoma cells. However, the great heterogeneity of the results suggests prior testing of the tumor cells in order to personalize the treatment.

## 1. Introduction

Niraparib (MK-4827) inhibits PARP1 and PARP2 [1]. It was approved by the U.S. Food and Drug Administration (FDA) in March 2017 indicated for the therapy of adult patients with ovarian, fallopian tube and peritoneal neoplasms [2]. Talazoparib (BMN 673) is a potent and selective inhibitor of PARP1 and PARP2 used at lower concentrations than previous generations of PARP inhibitors [3]. The FDA approved Talazoparib in October 2018 for patients with germline BRCA-mutated, HER2-negative breast cancer [4].

Poly (ADP-ribose) polymerases (PARP) play an essential role in different cellular processes, including several pathways of DNA repair [5,6,7]. PARP inhibitors (PARPi) are able to impair DNA damage repair by non-homologous end joining (NHEJ). This effect depends on the cell´s ability to compensate for the inhibition of PARP-mediated pathways by other repair pathways. PARPi especially induce cell death in cancer cells with a lack of PARP-independent DNA repair pathways. A known example of this synthetic lethality is the sensitivity of homologous recombinant (HR) deficient cells to PARPi [6,8]. Recently, this mechanism was confirmed in a clinical phase III trial using Olaparib in patients with homologous recombination deficient prostate cancers [9]. A further characteristic of PARPi is the so-called trapping of PARP. Trapped PARP does not dissociate from the lesion site and therefore blocks the damaged DNA position by complexation. This may reduce the possibility of completing DNA repair by other repair proteins [10]. Consequently, repair incompetence and accumulation of DNA lesions cause increased genomic instability and a higher probability of cell death [5,6].

Furthermore, PARPi treatment could be extended to cancer treatment in combination with DNA-damaging treatments [11]. The principle of enhancing the DNA-damaging effect of radiotherapy with simultaneous chemotherapy is widely known [12]. It has been suggested that PARPi offer the opportunity to enhance the efficacy of radiotherapy [7,13]. Noticeably, some DNA damage repair protein inhibitors targeting double-strand break repair proteins are also known to influence radiation therapy on a cellular level [14]. However, an increased risk of side effects after simultaneous therapy with kinase inhibitors and irradiation is described [15,16]. Based on commonly used melanoma cell lines Weigert/Jost et al. [17] have done a first overview study about the influence of the two PARPi Niraparib and Talazoparib in combination with ionizing radiation on melanoma cell cultures in vitro. With the intention of further specifying their experimental conditions, we focused on a patient-derived setting. Cell lines with nearly primary cell character were generated from surgical resections of melanomas.

## 2. Materials and Methods

### 2.1. Cell Cultures

The primary melanoma cell cultures ANST, ARPA, RERO, LIWE, ICNI and HV18MK were used for the experiments. The primary cells were established from human melanomas (primary tumors) in the Department of Dermatology of the University Hospital Erlangen. All tumor samples were obtained after the informed consent of the patients. The ethical approval was obtained from the Ethics Committee of the medical faculty of the Friedrich-Alexander-Universität Erlangen-Nürnberg (204_17 BC). The tissue samples were digested with collagenase and hyaluronidase (both substances Sigma Aldrich, München, Germany) as well as with DNAse (Roche, Mannheim, Germany) resulting in single-cell suspensions [18]. The cells were cultured for a maximum of 50 passages in RPMI-1640 medium (Sigma Aldrich, München, Germany). This medium was supplemented with 20% fetal bovine serum (FBS), 1% non-essential amino acids (NEA), 1% 1 M—Hepes buffer and 0.04% gentamicin (all four substances Merck, Darmstadt, Germany) as well as 1% L-glutamine and 1% pyruvate solution (both substances Gibco, Waltham, MA, USA). The primary fibroblasts SBLF7 and SBLF9 were derived from two different healthy individuals. The skin pieces were covered with a drop of F12 medium (Gibco, Waltham, MA, USA) supplemented with 40% FBS until they adhered to the culture flasks and the first fibroblasts had grown out [19]. Thereafter, the fibroblasts were covered with F12 medium supplemented with 12% FBS and 2% NEA as well as 1% penicillin-streptomycin (Gibco, Waltham, MA, USA). In the preparation phase for the experiments, the fibroblasts were grown in F12 medium supplemented with 15% FBS, 2% NEA and 1% penicillin-streptomycin. All cells were cultured in an incubator with a constant temperature of 37 °C and a humidified 5% CO_2_ atmosphere.

### 2.2. Inhibitors and Irradiation Treatment

Talazoparib and Niraparib (both Selleck Chemicals LLC, Huston, TX, USA) were stored at −80°C after producing aliquots of 1000 nM stock solution. For each experiment, a portion was thawed immediately before use. The concentrations of Talazoparib and Niraparib were chosen in accordance with the achievable plasma concentrations in humans. The maximum plasma concentration (cmax) of Talazoparib after repeated daily dosing of 1 mg is 16.4 ng/mL [20]. Due to the molecular weight of 380.4 g/mol, this corresponds to 43 nM [21]. The cmax of Niraparib is 804 ng/ml after giving a single dose of 300 mg [22], which corresponds to 2509 nM due to its molecular weight of 320.4 g/mol [1]. Based on these data, our experiments were conducted with concentrations of 50 nM Talazoparib respectively 2500 nM Niraparib. 

Cells were irradiated with a dose of 2 Gy ionizing radiation (IR) by an Isovolt TitanX-ray generator (GE Sensing and Inspection Technologies GmbH, Ahrensburg, Germany). For the combined treatment with an inhibitor plus IR, the irradiation was performed 3 h after the inhibitor treatment [23].

### 2.3. Analysis of the Relative PARP Expression

The relative PARP expression in melanoma cells compared to healthy fibroblasts was analyzed using the Western Blot assay. In a second pass, the melanoma cells were treated with a combination of the inhibitor Talazoparib respectively Niraparib and irradiation. The change of the PARP expression was determined 48 h after this treatment.

**Figure 1:**
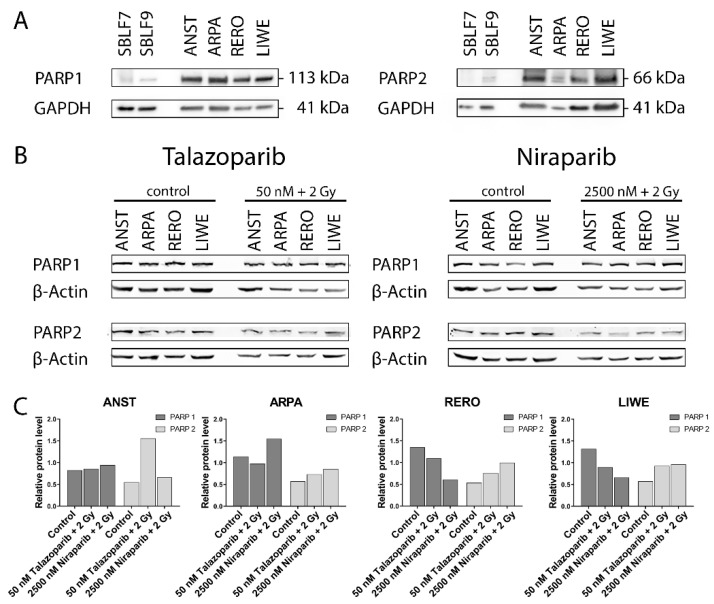
Western blot analyses of the PARP1 and PARP2 content. Levels of PARP1 and PARP2 in Western blot of (**A**) untreated healthy skin fibroblast and melanoma cell cultures and (**B**) melanoma cell cultures 48 h after the treatment with PARPi (50 nM Talazoparib respectively 2500 nM Niraparib) plus 2 Gy IR compared to untreated control groups. (**C**) Quantification of the PARP1 and PARP2 protein content after the combined treatment.

The cytoplasmic and the nuclear proteins of the cells were isolated separately. The nuclear fractions were used for the further procedures. The proteins were denatured at 95 °C and each sample was diluted to the same protein concentration of 1775 µg/mL. Subsequently, gel electrophoresis and Western blots were performed [24,25]. The primary antibodies anti-PARP1 (1:5000, rabbit, ab32138, Abcam, Cambridge, UK) or anti-PARP2 (1:1000, rabbit, ab176330, Abcam, Cambridge, UK) were used and incubated overnight at 4 °C. The secondary antibody goat anti-rabbit (1:15,000, ab6721, Abcam, Cambridge, UK) was incubated for 60 min at room temperature. The blots were developed by the iBright FL 1000 imager (Invitrogen, Thermo Fisher Scientific, Waltham, MA, USA). After that procedure, the blots were stripped. The loading control experiment was partially executed using the primary antibody anti-GAPDH (1:10,000, rabbit, ab181602, Abcam, Cambridge, UK) together with the secondary antibody anti-rabbit (1:15,000, goat, ab6721, Abcam, Cambridge, UK). Alternatively (Figure 1), the loading control process was executed using the primary antibody anti-β-Actin (1:10,000, mouse, ab6274, Abcam, Cambridge, UK) to avoid cancer-related bias as known for GAPDH in conjunction with the secondary antibody anti-mouse HRP (1:15,000, donkey, ab6820, Abcam, Cambridge, UK).

### 2.4. Flow Cytometry Analysis

Healthy fibroblast and melanoma cells were seeded in a 20% FBS medium to generate approximately a 50% confluence. Afterwards, the medium was replaced by a 2%-FBS medium. This reduced cell culture medium was used to not artificially enhance the effect of the inhibitor. The cells were divided into four groups: the first group stayed untreated, being used as a control group, the second one was treated with IR, the third one with an inhibitor (Talazoparib respectively Niraparib) and the fourth one with IR plus inhibitor (Talazoparib respectively Niraparib). All groups were incubated for 48 h. Subsequently, the adherent cells, as well as the non-adherent cells, were harvested. Each sample was bisected, the first half being used in a DNA cytometry test by Hoechst 33342 staining and the second half in an apoptosis/necrosis assay. The first half was placed in a mixture of cold 70% ethanol with FBS-reduced medium and was incubated at 4 °C for at least 12 h for fixation. Then these cells were stained with Hoechst 33342 (Invitrogen, Waltham, MA, USA) [26]. The second half of cells was stained immediately after the bisection with Annexin-V-APC and 7-amino-actinomycin D (both substances BD, Heidelberg, Germany) [27]. Both measurements were executed by fluorescence-activated cell sorting in the flow cytometer Cytoflex S (Beckman Coulter, Brea, CA, USA). The results were analyzed using the Kaluza analysis software (Beckman Coulter, Brea, CA, USA). 

### 2.5. Colony Formation Assay

Cells from healthy fibroblast and melanoma cell cultures were seeded in Petri dishes. After 14 hours of seeding, the cells were treated equivalently to the flow cytometry experiments. After 10 days, the cells were stained with methylene blue (#66725, Sigma Aldrich, München, Germany) for 30 min at room temperature. Stained colonies of at least 50 cells were visually selected and manually counted. The results of the cell line experiments are depicted in semi-logarithmic plots.

### 2.6. Homologous Recombination Assay (RAD51 Immunofluorescence Staining)

For proficiency analysis of homologous recombination, cells were seeded on cover slides to 90% confluence [14]. The cells were treated with CC-115 (5 μM; CAS No. 1228013-15-7) and subsequently irradiated (10 Gy dose) 24 h afterwards. The cells were fixed and permeabilized after 4 h (4% formaldehyde and 0.1% Triton ×−100/PBS). After blocking (1% bovine serum albumin; SERVA Electrophoresis GmbH, Heidelberg, Germany), the slides were stained with the primary antibodies mouse anti-γH2AX (1:1500, Merck, Darmstadt, Germany) and rabbit anti-Rad51 (1:250, abcam, Cambridge, UK) and with the secondary antibodies AlexaFluor488 goat anti-mouse and AlexaFluor594 chicken anti-rabbit (Invitrogen, Eugene, OR, USA). The nuclei were stained using DAPI (Sigma Aldrich, St. Louis, MO, USA). The image acquisition was performed by a Zeiss Axio Plan 2 fluorescence microscope (Zeiss, Göttingen, Germany) and an automated quantification was done using Biomass Software (MSAB, Erlangen, Germany).

### 2.7. Statistical Methods

Flow cytometry experiments and colony formation experiments were repeated at least three times. All results were analyzed by a two-sided Mann–Whitney-U-test using GraphPad Prism version 7.04 (GraphPad Software, Inc., San Diego, CA, USA). A *p*-value of ≤0.05 was determined to be statistically significant.

### 2.8. Biosecurity and Institutional Safety Procedures

Standard biosecurity and institutional safety procedures were followed.

### 2.9. Ethics Approval and Consent to Participate

Ethical approval was obtained in the Department of Dermatology, Universitätsklinikum Erlangen following approval by the institutional review board (Ethik-Kommission der Friedrich-Alexander-Universität Erlangen-Nürnberg, approval No. 204_17 Bc). The patients provided written informed consent.

## 3. Results

### 3.1. PARP1 and PARP2 Levels in the Cell Lines

The baseline PARP1 and PARP2 levels were higher in the four melanoma cell lines ANST, ARPA, RERO and LIWE compared to the two healthy skin fibroblast cell cultures SBLF7 and SBLF9 (Figure 1A). In the control groups of the four melanoma cell lines, ANST, ARPA, RERO and LIWE, the relative levels of PARP1 appeared to be higher than those of PARP2. The untreated control groups of the LIWE, RERO and ARPA cell lines tended to have a higher PARP1 level than the ANST cell line. Combined treatment of Talazoparib plus IR tended to decrease PARP1 in ARPA, RERO and LIWE cancer cell cultures. Similarly, after the application of Niraparib plus IR, the PARP1 level tended to decrease in the RERO and LIWE cell cultures. Contrary to that, in the ARPA cell culture, the PARP1 content seemed to increase after the combined treatment of Niraparib plus IR. ANST cell line did not change their PARP1 levels after treatment with inhibitor and IR. The PARP2 expression in the untreated control groups of all four tested melanoma cell cultures indicated similar levels. A combined treatment of these melanoma cell cultures with Talazoparib respectively Niraparib plus IR tended to increase the PARP2 content (Figure 1B,C). Furthermore, data were then normalized to β-Actin to avoid possible bias of different GAPDH expression which is known for different cancer cells. To summarize, a combined treatment of PARP inhibitor and IR primarily resulted in a decrease or a constant level of PARP1 content, whereas the PARP2 content increased in all four melanoma cell lines. No statistical significance could be derived from our experiments, but nevertheless, interesting tendencies emerged (Figure S1).

### 3.2. Induction of Apoptosis and Necrosis by the PARPi

**Figure 2:**
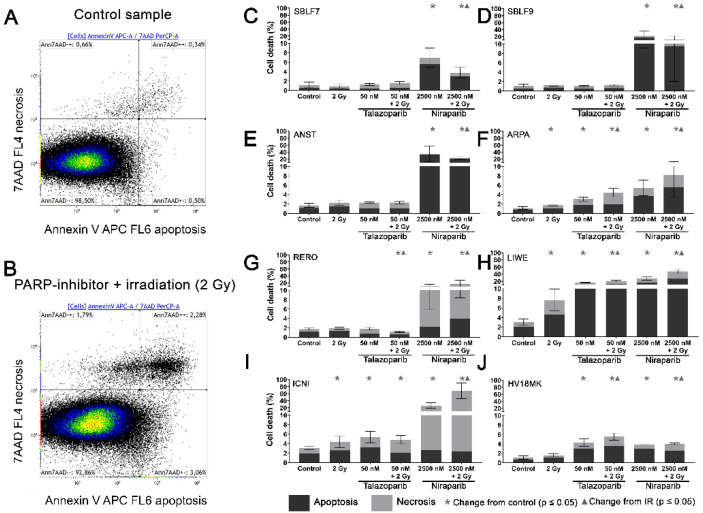
Apoptotic and necrotic rates after combined PARPi plus IR treatment. Healthy skin fibroblast and melanoma cell cultures were analyzed 48 h after the treatment with IR, with a PARPi Talazoparib (50 nM) respectively Niraparib (2500 nM), with IR plus an inhibitor or without treatment with regard to their apoptotic and necrotic rates. (**A**) Control sample and (**B**) a sample after combined treatment of PARPi plus IR in the flow cytometer analysis of Annexin/7-AAD staining. (**C–J**) Apoptotic and necrotic rates in eight different cell cultures. A logarithmic scaling was chosen for a better representability. The data were analyzed using the Mann–Whitney-U test. (All %-values and *p*-values of cell death are included in the supplementary material Tables S1–S3).

Two characteristic examples of the Kaluza analysis of our apoptosis and necrosis experiments are displayed (Figure 2A,B). Most of the cells were neither Annexin nor 7-AAD positive and therefore identified as living cells. This cluster of living cells is depicted in the lower-left quadrant of the chart. After combined treatment with PARP inhibitor plus IR, the cells slightly increased in the lower right Annexin-positive quadrant, representing apoptosis. The Annexin plus 7-AAD-positive upper right quadrant represents necrosis (Figure 2B). The combined treatment is compared to the untreated controls (Figure 2A).

In the subsequent analysis, apoptosis and necrosis are summarized as cell death. In the healthy fibroblast cell lines SBLF7 respectively SBLF9 (Figure 2C,D), neither the IR treatment nor the Talazoparib mono-treatment nor the combined treatment of Talazoparib plus IR led to a significant change in the cell death rate compared to the control groups. However, in both fibroblast cell cultures, the Niraparib treatment significantly increased cell death. Likewise, the combined treatment of Niraparib plus IR increased cell death (*p* = 0.036 respectively *p* = 0.048). In the cancer cell lines (Figure 2E–J), the rates of cell death induction varied distinctly more than in the healthy fibroblasts. After IR treatment, cell death increased clearly in ARPA, LIWE and ICNI melanoma cell lines (*p* ≤ 0.05) (Figure 2F,H,I). The IR treatment did not noticeably induce cell death in ANST, RERO and HV18MK melanoma cell lines (Figure 2E,G,J). Talazoparib treatment did increase the rate of cell death in ARPA, ICNI, HV18MK and most in LIWE cell lines (*p* ≤ 0.05) (Figure 2F,I,J,H). After a combined treatment of Talazoparib plus IR, the cell death increased between 2% and 18% in ICNI, ARPA, HV18MK and again most in LIWE (*p* ≤ 0.05) (Figure 2I,F,J,H). Furthermore, a significant increase in cell death compared to the IR mono-treatment was observable in ARPA, HV18MK and LIWE, varying between 3% and 13% (*p* ≤ 0.05). After combined Talazoparib plus IR treatment, however, cell death decreased slightly in the RERO cell culture compared to the untreated control group as well as to the IR mono-treatment (Figure 2G). In all melanoma cell cultures, the Niraparib treatment and the combined treatment of Niraparib plus IR again led to an increase in cell death. This increase was 3% in HV18MK as well as 65% in ICNI. The increases in the other four melanoma cell lines were scattered between these two values (*p* ≤ 0.05). In addition, the combined treatment of Niraparib plus IR increased cell death compared to the IR mono-treatment, varying between a 2% increase in HV18MK and a 64% increase ICNI (*p* ≤ 0.05).

The difference between the sum of the particular mono-treatments and the corresponding combined treatment was calculated to obtain PARPi and IR interactions. The underlying values of the logarithmic graphs in Figure 2C–J were used. Sub-additive effects were observed in the SBLF9 and the ANST cell lines after the combined treatment of Niraparib plus IR (SBLF9: −10%; ANST: −12%). In contrast, supra-additive effects were observed in the LIWE and the ICNI cell lines after the combination of Niraparib plus IR (LIWE: +12%; ICNI: +38%). For the remaining cell lines, the effects were approximately additive (Tables S1–S6).

### 3.3. Cell Cycle G2/M Arrest

**Figure 3:**
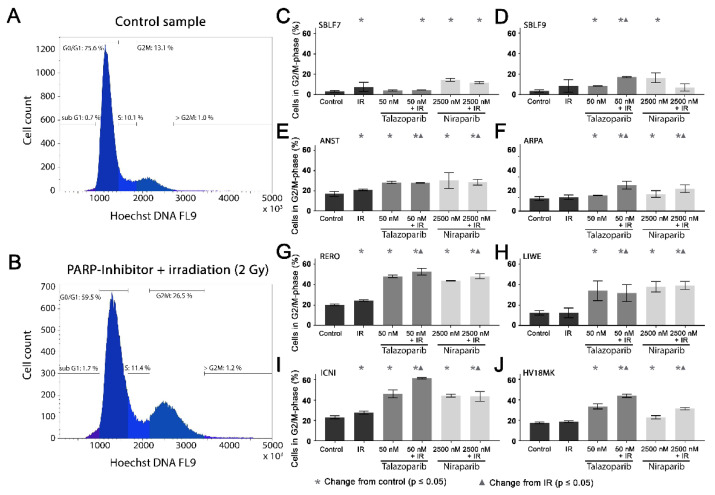
G2/M phase arrest after combined PARPi plus IR treatment. The G2/M phase content of healthy skin fibroblast and melanoma cell cultures. The DNA content was analyzed by flow cytometry of Hoechst 33342 staining 48 h after the treatment with IR, with a PARPi (50 nM Talazoparib respectively 2500 nM Niraparib) or with IR plus an inhibitor. The control groups stayed untreated. (**A**) Histogram of the cells in the different cell cycle phases of a control sample. (**B**) A sample after a combined treatment of PARPi plus IR. (**C–J**) G2/M phase fraction in eight different cell cultures. The data were analyzed using the Mann–Whitney-U test. (All %-values and *p*-values of cell cycle are included in the supplementary material Tables S4–S6).

Cell cycle arrest in the G2/mitosis (G2/M) phase was studied using Hoechst 33342 DNA staining. The DNA content was graphically visualized (Figure 3A,B). The first peak represents the G0/G1 phase cells with single DNA content and the second peak represents the G2/M phase cells with double DNA content. The S phase cells are in between the two peaks (Figure 3A). A combined treatment leads to an increase in the G2/M phase (Figure 3B).

In the healthy fibroblasts SBLF7 and SBLF9, the G2/M phase cells accounted for 3% (Figure 3C,D). IR treatment increased the G2/M cell cycle arrest of the fibroblasts. After the combined treatment of Talazoparib plus IR, the G2/M fraction of the two healthy fibroblast cell lines did increase (*p* ≤ 0.05). Niraparib mono-treatment did increase the G2/M fraction, whereas the combined treatment tended to reduce this effect.

The G2/M levels in the melanoma cell lines (Figure 3E–J) were distinctly higher than in the fibroblasts, varying between 12% in the ARPA and 23% in the ICNI cell cultures. In the treated groups, G2/M arrest increased in the melanoma cell cultures more than in the fibroblast cultures. IR treatment did increase the G2/M fraction in the ANST, RERO and ICNI cultures (Figure 3E,G,I). After treatment with one of the inhibitors as well as after combined treatment with one inhibitor plus IR, the arrest in G2/M did increase in all melanoma cell cultures: 3% in ARPA up to 38% in ICNI, with the values of the other four melanoma cell lines scattering in between (*p* ≤ 0.05). Compared to the IR mono-treatment, the combined treatment with one inhibitor plus IR did increase the G2/M arrest values in all melanoma cell cultures, varying between 7% in ANST and 34% in ICNI (*p* ≤ 0.05). There are large differences in G2/M arrest between the different cell lines. Additionally, we analyzed the subG1 phase for comparison with our cell death FACS analysis (Figure S2).

### 3.4. Surviving Fraction

**Figure 4:**
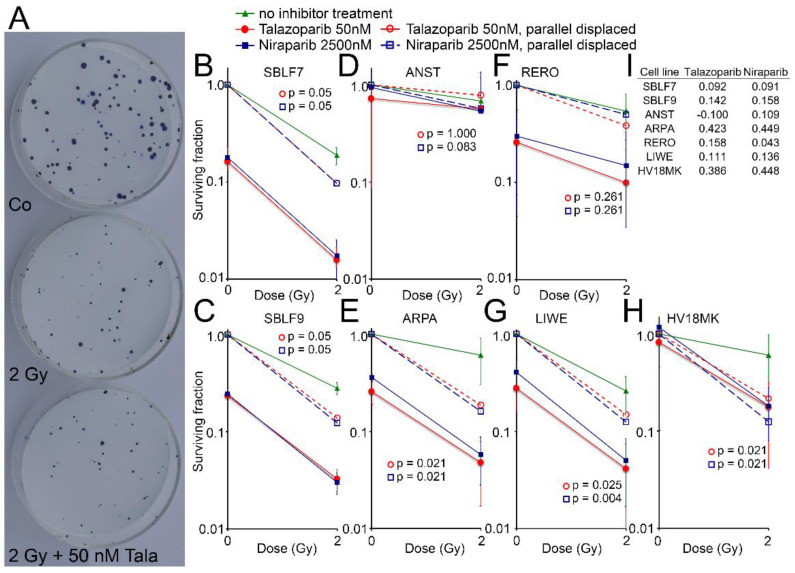
Colony formation assay after combined PARPi plus IR treatment. (**A**) Representativ colony formation assay in petri dishes of skin cancer cell line RERO untreated (Co), irradiated (2 Gy) and after combined therapy (2 Gy + 50 nM Talazoparib). (**B–H**) Surviving fraction after 0 and 2 Gy IR and after treatment with the PARPi Talazoparib (50 nM, red lines) respectively Niraparib (2500 nM, blue lines) (Solid lines). Normalized values for the effect of the inhibitors (stripped lines). (**I**) The difference between the parallel displaced values of the combined effect and the radiation effect alone indicating the increased effect of the combined treatment.

Cell surviving was observed for the two healthy fibroblasts and for five melanoma cell lines by the colony formation assay. Images of Petri dishes from a control sample and after treatment are shown (Figure 4A). The healthy fibroblast cultures SBLF7 and SBLF9 (Figure 4B,C) were clearly sensitive against the inhibitors themselves and even more against the combined treatment of an inhibitor plus IR. The melanoma cell lines ARPA, RERO and LIWE were similarly sensitive (Figure 4E–G). The HV18MK melanoma cell culture was apparently less sensitive against the combined treatment and not sensitive against the inhibitors (Figure 4H). The ANST melanoma cell culture was neither sensitive against the inhibitors nor the combined treatment (Figure 4D).

The curves of the combined treated cell lines were shifted parallel to display the interaction between the inhibitors and irradiation. The differences of the parallel displaced combined treatment to IR treatment alone are listed in Figure 4I, representing a deviation from a supposed additive effect. In the cell lines ARPA and HV18MK, a pronounced supra-additive effect was observed for both inhibitors (*p* ≤ 0.05). For the fibroblasts and the melanoma cell cultures ANST, RERO and LIWE, there were minor supra-additive effects and in the case of ANST, there were none for Talazoparib.

### 3.5. Homologous Recombination Status

Additionally, we analyzed the homologous recombination (HR) status of the cell lines (Figure S3). The healthy fibroblasts SBLF7 and SBLF9 as well as the melanoma cell lines ANST and LIWE were HR-proficient, which was indicated by an increase of the RAD51 foci. RAD51 foci decreased in the melanoma cell cultures ARPA, RERO, ICNI and HV18MK and therefore these cell lines were defined as HR-deficient.

## 4. Discussion

Due to a growing interest in PARPi for cancer therapy, the development of such substances and the further extension of their application have progressed in recent years [9]. Since ionizing radiation leads to DNA damage, the combining of radiotherapy and inhibitors of proteins essential for DNA damage repair, like PARPi Talazoparib and Niraparib, appears to be beneficial. Hence, we studied the combined effects of these two PARPi and subsequent ionizing radiation in vitro. Because Talazoparib and Niraparib act via binding to their specific targets PARP1 and PARP2, we assumed that differences in the expression level of the targets may explain differences in the response to the inhibitors. In this regard, PARP1 appears to be the main target of PARPi [13]. One reason for this could be that PARP1 contributes the largest proportion of the total PARP activity, whereas that of PARP2, which is less abundant, is only about 5% to 10% [28]. We found that the protein levels of PARP1 and PARP2 were higher in the melanoma cells compared to the healthy skin fibroblasts. Similar results for an increased expression of PARP1 in tumor cells had already been published [11]. Thereby, the authors expected potential for increased sensitivity to PARPi in cancer treatment. In our studies, we found hints for correlations between PARP1 content and sensitivity to PARPi: among the melanoma cell lines tested, ANST had the lowest relative PARP1 level as well as the lowest sensitivity against both inhibitors in the colony formation experiments. However, following our further cell death analysis, we cannot unambiguously agree to the above-mentioned hypothesis of a correlation between PARP content and sensitivity to PARPi. Thus, we agree with other authors, who postulate that the levels of the PARP enzymes in tumor tissue are no valid biomarkers for the clinical response and resistance in patients [29]. Additional tests, including the DNA damage repair capacity of the tumor cells, are recommended [7]. 

A comparison of cell death and clonogenic survival can be made based on the Annexin/7-AAD stain and colony formation assays. Clonogenic survival considers additional types of cell death such as mitosis-coupled cell death and the additional loss of clonogenic survival through senescence. Talazoparib treatment had no effect on cell death of the healthy skin fibroblasts. In contrast, Niraparib treatment, with a markedly higher concentration than Talazoparib, according to the higher concentration in clinical use, had a clear effect: cell death increased compared to the untreated control group and compared to the IR mono-treatment. However, in the clonogenic survival assays, both drugs indicated a very pronounced effect in the healthy fibroblasts, which further increased by additional IR. The cause for the strong effects in clonogenic survival is probably the induction of senescence, which is very common in fibroblasts [30]. Similarly, in melanoma cell lines the drug alone caused a significant loss of clonogenic survival, which is probably caused by the induction of senescence as well [31].

Regarding cell death, two remarkable supra-additive effects were found after the combination of Niraparib and IR. On the contrary, two sub-additive effects were noticeable in the SBLF9 and ANST. Thus, the PARP inhibitor Niraparib in particular seems to interact with IR treatment exceptionally divergent. Since clonogenic survival is the gold standard in radiation biology it is of remarkable interest that 13 out of 14 KI+IR combinations were able to decrease survival in a supra-additive manner. This gives clear hints to the benefit of concomitant radiotherapy and targeted therapy of the DNA repair system. Overall there were clear differences between cell death and loss of clonogenicity. The loss of clonogenicity is probably the more relevant variable to make a prediction for use in radiochemotherapy [32,33]. It points out that most melanoma cells are sensitive to PARPi and that the combined treatment with ionizing radiation might be promising for patients with melanoma.

Previous studies reported a G2/M cell cycle arrest after PARPi [34,35,36]. An enhancement of this effect by combination of PARP1 inhibition with irradiation compared to monotherapy was described [37]. Our studies confirm these observations regarding melanoma cells. Since the G2/M phase is the most radiation-sensitive cell cycle phase [38], the G2/M arrest could be the reason for the radiosensitization of melanoma cells [39]. Especially in the case of fractionated radiation therapy, the following IR fraction then hits the particularly radiation-sensitive cells in the G2/M phase more frequently [40]. The surrounding healthy tissue would then be less affected by the radiosensitization due to the lower cell count in the G2/M phase. Nevertheless, the shifts to the G2/M phase do not correlate one-to-one with the results from the cell death experiments. We have to assume that the correlations are significantly more complex, which will be briefly discussed in the following paragraphs.

The effects of the PARPi Talazoparib onto the cell lines partially differed significantly from the effects of Niraparib onto identical cell lines. The comparison of cell death analysis and the colony formation assay sheds light on the assumption that Talazoparib triggers senescence and clonogenicity loss in cells rather than cell death. On the other hand, the application of Niraparib indicates increased cell death. An additional analysis of the subG1 phase showed corresponding results (Figure S2). The emerging differences in the effects of the inhibitors on the different cell lines were particularly striking. Similar observations were previously made by Weigert/Jost et al. [17]. Therefore, in this study, we assembled a cell line panel that had a primary cell character to generate a more patient-linked setting. In addition, we assembled colony formation assays as the gold standard in radiation biology to compare with the cell death experiments. The results of the colony formation assays were quite different from the results of the cell death experiments. Induction of senescence by PARPi, in addition to cell death, is another possibility for limited clonogenicity in the colony formation assay. Senescence is a further important mechanism to remove tumor cells from the proliferation cycle [41].

Another possible cause for these differences in the cell lines could be variations in the function of the HR. Therefore, we analyzed the homologous recombination (HR) status of our cell lines to get a better understanding of the inter-cellular differences (Figure S3). An HR proficiency could be the reason for the low response to the treatment in the colony formation assay, like our data suggested for the HR-proficient cell line ANST. Even though we found interesting new explanatory approaches for the heterogeneous effects, we are not yet able to fully explain all of our observations made. Our studied cell line panel represents a broad spectrum of different melanoma patients and likely contains a diverse profile of mutations. Deeper analyzes and genetic characterizations seem necessary to gain a better understanding of the mutations of individual tumor cells. This could help to establish clearer correlations about the effects of the inhibitors, especially also in combination with irradiation. Ideally, this would lead to safer recommendations of a combined PARPi and radiation therapy for individual melanoma patients in the future. 

Nevertheless, when thinking about future clinical development of such combination therapy, it is of great importance to consider the possibility of side effects. It is known that the ability of kinase inhibitors to radiosensitize cells carries the risk of side effects. In 2012, the first patient suffered from radio-dermatitis after concomitant radiotherapy and treatment with the BRAF inhibitor Vemurafenib. Hecht et al. [15] described the radiosensitizing effect of Vemurafenib and appearing side-effects of concomitant therapy in melanoma patients. Interaction-related side effects could also be possible for our two PARPi in combination with radiation.

Several ongoing trials of Talazoparib (e.g., NCT04170946, NCT03968406) and Niraparib (e.g., NCT04037254, NCT03644342) in combination with radiation may also yield interesting results regarding potential side effects. The ongoing clinical trials confirm the relevance of the consideration, whether combining Talazoparib respectively Niraparib with radiation could potentially benefit cancer treatment.

## 5. Conclusions

We found that most of the melanoma cell lines studied were sensitive to the combined treatment with inhibitors and irradiation with regard to cell survival: four out of six cell lines were sensitive to Talazoparib plus IR treatment and six out of six cell lines were sensitive to Niraparib plus IR treatment. These results were even more pronounced when considering clonogenic survival: four out of five cell lines were sensitive to both inhibitors in combination with irradiation treatment. In addition, we found a clear potential for radiosensitization of melanoma cells with both inhibitors in combination with irradiation: in six out of six cell lines, the proportion of cells in the G2/M phase increased. This radiosensitization could increase the efficiency in clinical radiotherapy of melanoma patients. In addition, the less radiation-sensitive healthy tissue would be spared during therapy. However, we could not establish a general benefit of the combined therapy, since individual melanoma cell lines are resistant to the therapy. Therefore, an individualization of the therapy with prior testing of the tumor tissue should be the first step towards efficient combination therapy.

## Supplementary Materials

The following are available online at https://www.mdpi.com/article/10.3390/genes12060849/s1, Figure S1: Western blot analysis of three individual experiments of PARP1 and PARP2 expression in four skin cancer cell lines. (A) Relative expression level of PARP1 in untreated ANST, ARPA, RERO and LIWE cells in three independent experiments. (B) Relative expression level of PARP2 in untreated ANST, ARPA, RERO and LIWE cells in three independent experiments. (C) Relative protein level of PARP1 and PARP2 in four skin cancer cell lines. Values represent three independent experiments (mean ± SD). Statistical significance was calculated by Mann–Whitney-U test and *p* ≤ 0.05 (*) was set as significant. (D) PARP1 and PARP2 expression in healthy fibroblasts SBLF7 in an untreated control, after 2 Gy IR, monotherapy of Talazoparib (50 nM), Niraparib (2500 nM) and combination therapy of KI + IR., Figure S2: Proportion of six skin cancer cell lines in subG1 phase (ANST, ARPA, LIWE, ICNI, RERO and HV18MK) and of two healthy donor fibroblasts (SBLF7, SBLF9). (A) Gating strategy to exclude cell duplets. Representative histograms of Hoechst-staining of SBLF9 fibroblasts under niraparib, irradiation or combination therapy. Distribution of cell cycle phases and gating of subG1 phase as an indicator for apoptotic bodies (apoptosis). (B) Proportion of six skin cancer cell lines and healthy fibroblasts in subG1 phase as an indicator for apoptosis. Cells were treated with Talazoparib (50 nM), Niraparib (2500 nM), irradiation (2 Gy) or combination therapy for 48 h., Figure S3: Efficiency of homologous recombination, a double-strand break repair pathway, in the skin cancer panel and both healthy controls. Analysis of γH2AX and Rad51 foci after a dose of 10 Gy IR and treatment with the DNA-PK inhibitor CC-115, an inhibitor of the non-homologous end-joining pathway. Compared to the untreated control, Rad51 foci increased upon IR in SBLF7, SBLF9, ANST and LIWE. Therefore, these four cell lines are considered HR-proficient. Rad51 foci decreased in skin cancer cell lines ICNI, RERO, ARPA and HV18MK which indicates HR deficiency. Table S1: Cell death in %. Table S2: Cell death: significant change to Control in %. Table S3: Cell death: significant change to IR in %. Table S4: Cell fraction in G2/M phase in %. Table S5: Cell fraction in G2/M phase: significant change to Control in %. Table S6: Cell fraction in G2/M phase: significant change to IR in %.
