# Supplementary material for: PARP Inhibitors Talazoparib and Niraparib Sensitize Melanoma Cells to Ionizing Radiation
Source: Genes (Basel). 2021 May 31;12(6):849. doi: 10.3390/genes12060849 (PMC8229922; doi:10.3390/genes12060849)
Supplement: Supplementary file 1 [file genes-12-00849-s001.zip › genes-1236971-supplementary.pdf]

PARP inhibitors Talazoparib and Niraparib sensitize melanoma cells to ionizing radiation

Stephanie Jonuscheit[1]*, Tina Jost[1]*, Fritzi Gajdošová[1], Maximilian Wrobel[1], Markus Hecht[1], Rainer Fietkau[1], Luitpold Distel[1]

1 Department of Radiation Oncology, University Hospital Erlangen, Germany

*These authors contributed equally to this work.

Supplementary Material

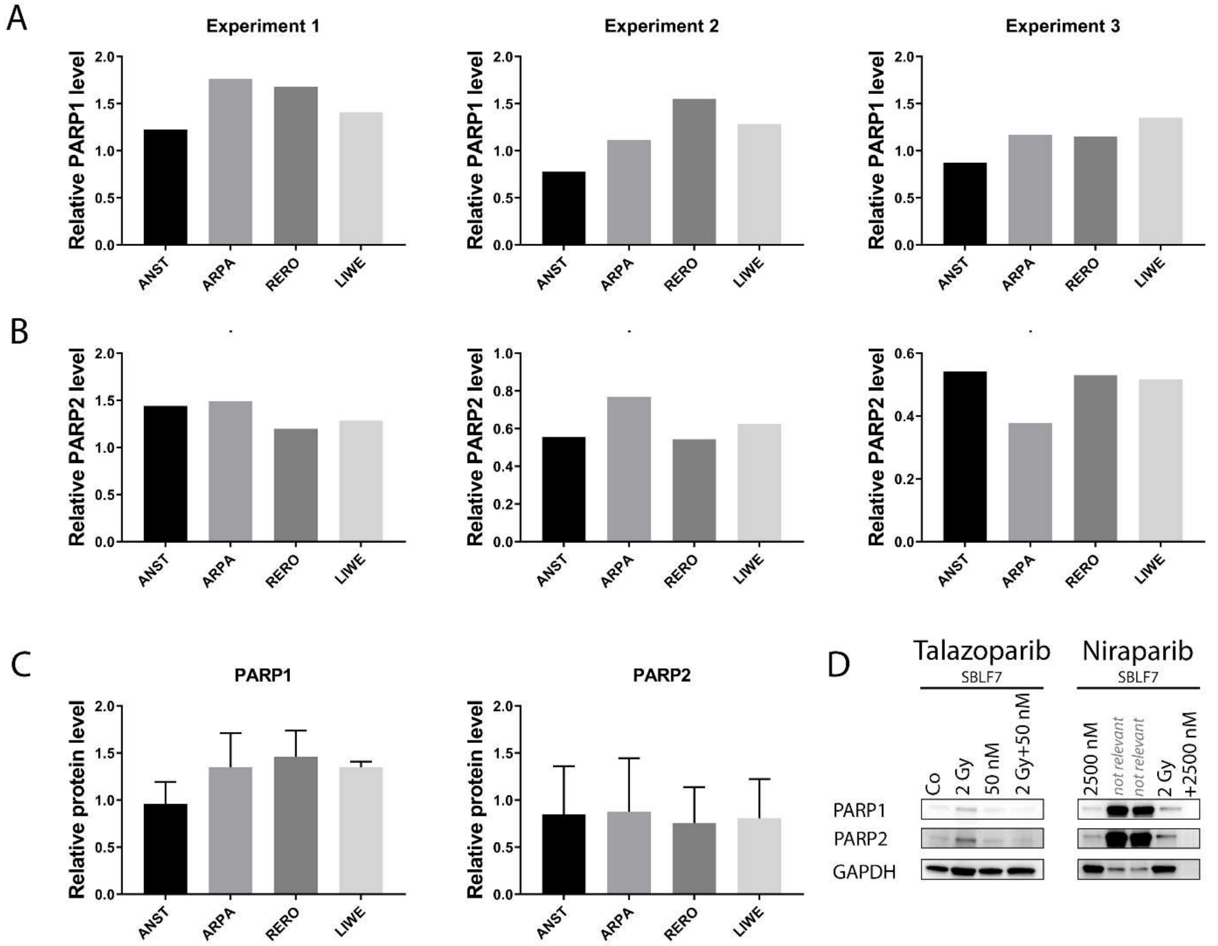

Figure S1: Western blot analysis of three individual experiments of PARP1 and PARP2 expression in four skin cancer cell lines. A) Relative expression level of PARP1 in untreated ANST, ARPA, RERO and LIWE cells in three independent experiments. B) Relative expression level of PARP2 in untreated ANST, ARPA, RERO and LIWE cells in three independent experiments. C) Relative protein level of PARP1 and PARP2 in four skin cancer cell lines. Values represent three independent experiments (mean ± SD). Statistical significance was calculated by Mann-Whitney-U test and $p \leq 0.05$ (*) was set as significant. D) PARP1 and PARP2 expression in healthy fibroblasts SBLF7 in an untreated control, after 2 Gy IR, monotherapy of Talazoparib (50 nM), Niraparib (2500 nM) and combination therapy of KI + IR.

PARP inhibitors Talazoparib and Niraparib sensitize melanoma cells to ionizing radiation

Stephanie Jonuscheit[1]*, Tina Jost[1]*, Fritzi Gajdošová[1], Maximilian Wrobel[1], Markus Hecht[1], Rainer Fietkau[1], Luitpold Distel[1]
1 Department of Radiation Oncology, University Hospital Erlangen, Germany
*These authors contributed equally to this work.

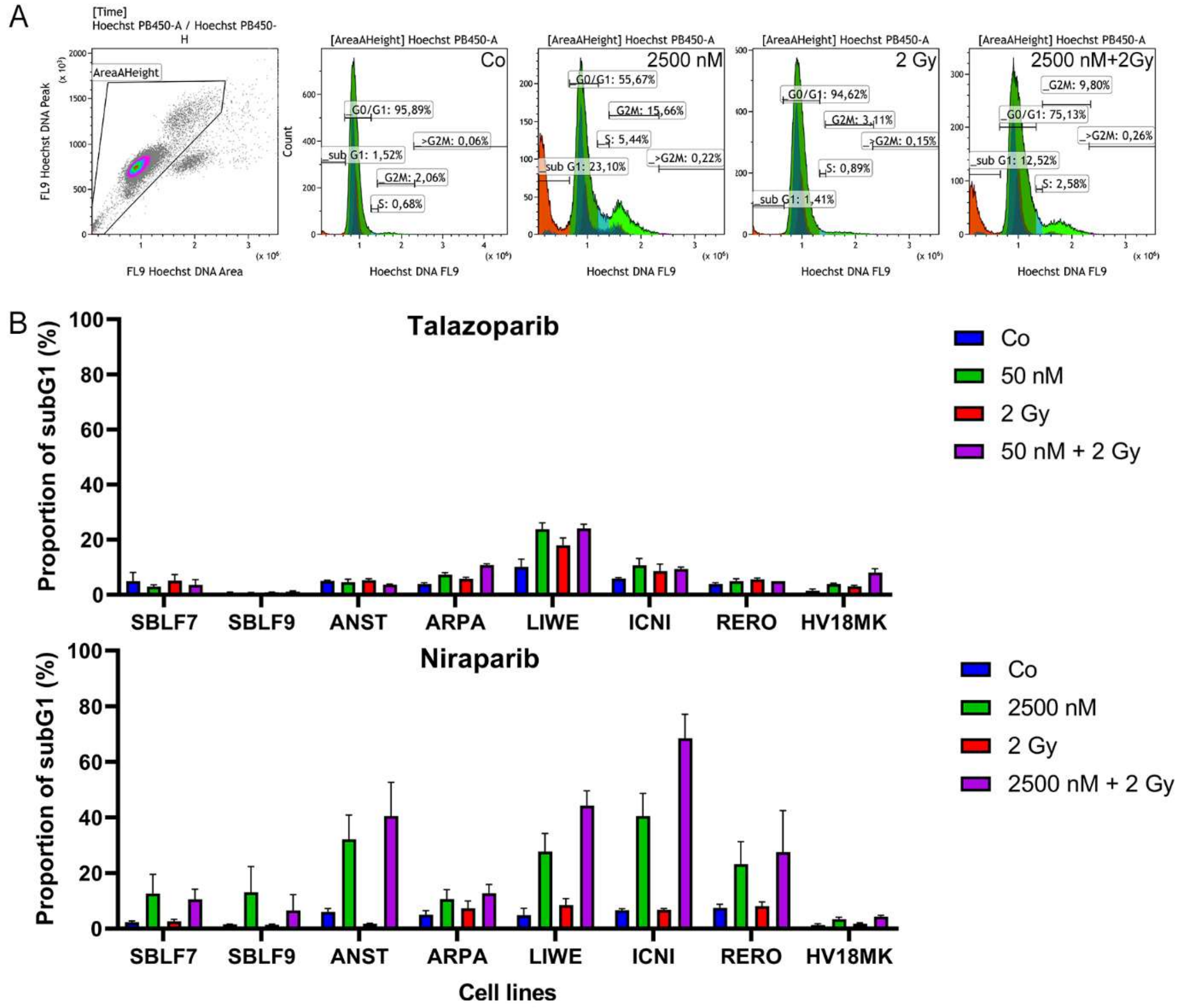

Figure S2: Proportion of six skin cancer cell lines in subG1 phase (ANST, ARPA, LIWE, ICNI, RERO and HV18MK) and of two healthy donor fibroblasts (SBLF7, SBLF9). A) Gating strategy to exclude cell duplets. Representative histograms of Hoechst-staining of SBLF9 fibroblasts under niraparib, irradiation or combination therapy. Distribution of cell cycles phases and gating of subG1 phase as indicator for apoptotic bodies (apoptosis). B) Proportion of six skin cancer cell lines and healthy fibroblasts in subG1 phase as indicator for apoptosis. Cells were treated with Talazoparib (50 nM), Niraparib (2500 nM), irradiation (2 Gy) or a combination therapy for 48 h. Values represent mean ± SD (n = 3).

PARP inhibitors Talazoparib and Niraparib sensitize melanoma cells to ionizing radiation

Stephanie Jonuscheit[1]*, Tina Jost[1]*, Fritzi Gajdošová[1], Maximilian Wrobel[1], Markus Hecht[1], Rainer Fietkau[1], Luitpold Distel[1]
1 Department of Radiation Oncology, University Hospital Erlangen, Germany
*These authors contributed equally to this work.

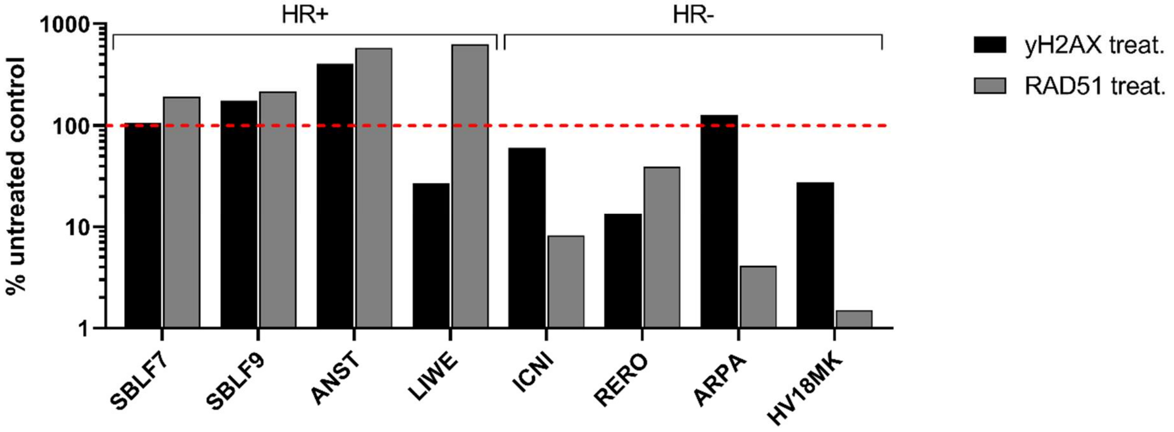

Figure S3: Efficiency of homologous recombination, a double strand break repair pathway, in the skin cancer panel and both healthy controls. Analysis of γH2AX and Rad51 foci after a dose of 10 Gy IR and treatment with the DNA-PK inhibitor CC-115, an inhibitor of the non-homologous end joining pathway. Compared to the untreated control, Rad51 foci increased upon IR in SBLF7, SBLF9, ANST and LIWE. Therefore, these four cell lines are considered as HR-proficient. Rad51 foci decreased in skin cancer cell lines ICNI, RERO, ARPA and HV18MK which indicates a HR-deficiency.

PARP inhibitors Talazoparib and Niraparib sensitize melanoma cells to ionizing radiation

Stephanie Jonuscheit[1]*, Tina Jost[1]*, Fritzi Gajdošová[1], Maximilian Wrobel[1], Markus Hecht[1], Rainer Fietkau[1], Luitpold Distel[1]
1 Department of Radiation Oncology, University Hospital Erlangen, Germany
*These authors contributed equally to this work.

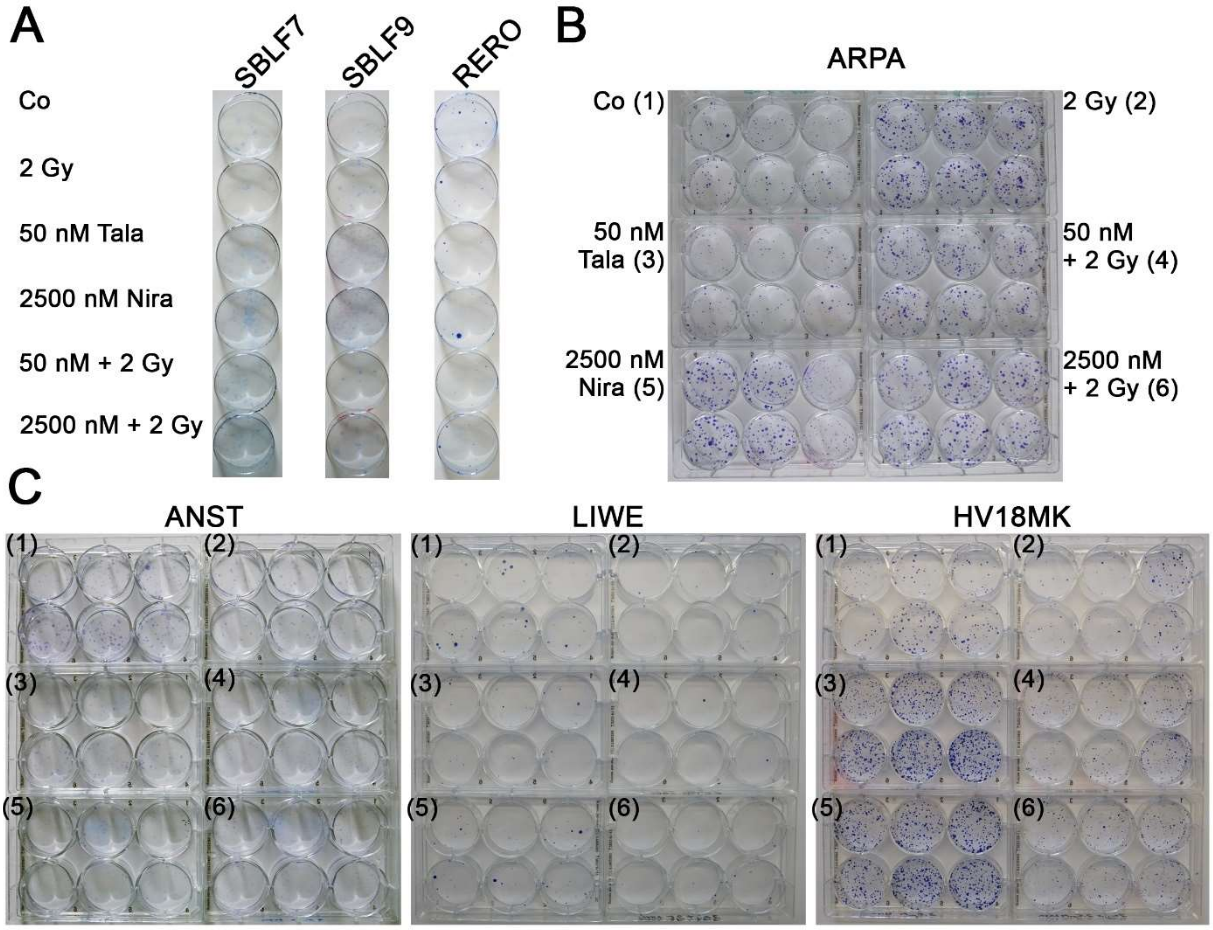

Figure S4: Representative images of colony forming assays of two healthy fibroblasts and four skin cancer cell lines. A) SBLF7, SLBF9 and RERO seeded in petri-dishes. Without treatment (Co), with irradiation (2 Gy), with kinase inhibitor Talazoparib (50 nM) and Niraparib (2500 nM) and a combination of both Tala+IR and Nira+IR. B) Skin cancer cells ARPA seeded in 6-well-plates. Without treatment (Co), with irradiation (2 Gy), with kinase inhibitor Talazoparib (50 nM) and Niraparib (2500 nM) and a combination of both Tala+IR and Nira+IR. C) Skin cancer cells ANST, LIWE and HV18MK seeded in 6-well-plates and treated according to cell line ARPA (1: Co, 2: 2 Gy IR, 3: 50 nM Talazoparib, 4: 50 nM Talazoparib + 2 Gy IR, 5: 2500 nM Niraparib, 6: 2500 nM Niraparib + 2 Gy IR. Images B) and C) represent 6 technical replicates.

PARP inhibitors Talazoparib and Niraparib sensitize melanoma cells to ionizing radiation

Stephanie Jonuscheit[1]*, Tina Jost[1]*, Fritzi Gajdošová[1], Maximilian Wrobel[1], Markus Hecht[1], Rainer Fietkau[1], Luitpold Distel[1]
1 Department of Radiation Oncology, University Hospital Erlangen, Germany
*These authors contributed equally to this work.

Analysis of the flow cytometry experiments

Cell death experiments

Table S1: Cell death in %.

	Control	IR	Talazoparib	Talazoparib + IR	Niraparib	Niraparib + IR
SBLF7	1.2	0.9	1.3	1.6	7.0	3.8
SBLF9	1.2	1.3	1.0	1.2	22.3	11.8
ANST	1.7	2.3	2.3	2.3	35.0	22.8
ARPA	1.1	1.7	3.1	4.4	5.4	8.1
RERO	1.7	1.9	1.8	1.2	11.0	18.0
LIWE	3.0	7.6	16.1	20.8	27.6	47.8
ICNI	3.1	4.4	5.4	4.8	26.5	68.4
HV18MK	1.1	1.6	4.3	5.6	3.9	4.0

Table S2: Cell death: significant change to Control in %.

	IR	Talazoparib	Talazoparib + IR	Niraparib	Niraparib + IR
SBLF7	-	-	-	5.8<br>p = 0.0357	2.6<br>p = 0.0357
SBLF9	-	-	-	21.1<br>p = 0.0238	10.6<br>p = 0.0476
ANST	-	-	-	33.3<br>p = 0.0238	21.1<br>p = 0.0238
ARPA	0.6<br>p = 0.0043	2.0<br>p = 0.0238	3.3<br>p = 0.0238	4.3<br>p = 0.0238	7.0<br>p = 0.0238
RERO	-	-	-0.5<br>p = 0.0357	9.3<br>p = 0.0238	16.3<br>p = 0.0238
LIWE	4.6<br>p = 0.0022	13.1<br>p = 0.0238	17.8<br>p = 0.0238	24.6<br>p = 0.0238	44.8<br>p = 0.0238
ICNI	1.3<br>p = 0.0087	2.3<br>p = 0.0476	1.7<br>p = 0.0476	23.4<br>p = 0.0238	65.3<br>p = 0.0238
HV18MK	-	3.2%<br>p = 0.0238	4.5%<br>p = 0.0238	2.8%<br>p = 0.0238	2.9%<br>p = 0.0238

Table S3: Cell death: significant change to IR in %.

	Talazoparib + IR	Niraparib + IR
SBLF7	-	2.9 p = 0.0238
SBLF9	-	10.5 p = 0.0476
ANST	-	20.5 p = 0.0238
ARPA	2.7 p = 0.0238	6.4 p = 0.0238
RERO	-0.7 p = 0.0238	16.1 p = 0.0238
LIWE	13.2 p = 0.0238	40.2 p = 0.0238
ICNI	-	64.0 p = 0.0238
HV18MK	4.0 p = 0.0238	2.4 p = 0.0238

PARP inhibitors Talazoparib and Niraparib sensitize melanoma cells to ionizing radiation

Stephanie Jonuscheit[1]*, Tina Jost[1]*, Fritzi Gajdošová[1], Maximilian Wrobel[1], Markus Hecht[1], Rainer Fietkau[1], Luitpold Distel[1]
1 Department of Radiation Oncology, University Hospital Erlangen, Germany
*These authors contributed equally to this work.

Cell Cycle experiments

Table S4: Cell fraction in G2/M phase in %.

	Control	IR	Talazoparib	Talazoparib + IR	Niraparib	Niraparib + IR
SBLF7	3.0	7.5	4.0	4.5	14.4	11.8
SBLF9	3.4	8.4	8.3	17.0	16.3	6.9
ANST	17.0	21.1	28.3	27.8	30.3	28.6
ARPA	12.4	13.6	15.4	25.5	16.8	22.0
RERO	20.2	24.5	48.0	52.6	43.6	48.1
LIWE	12.3	12.4	34.0	31.7	37.9	39.2
ICNI	23.2	27.8	46.1	61.4	44.3	43.6
HV18MK	17.4	18.7	33.6	44.0	22.9	31.4

Table S5: Cell fraction in G2/M phase: significant change to Control in %.

	IR	Talazoparib	Talazoparib + IR	Niraparib	Niraparib + IR
SBLF7	4.5<br>p = 0.0455	-	1.5<br>p = 0.0238	11.4<br>p = 0.0238	8.8<br>p = 0.0238
SBLF9	-	4.9<br>p = 0.0238	13.6<br>p = 0.0238	12.9<br>p = 0.0238	-
ANST	4.1<br>p = 0.0022	11.3<br>p = 0.0238	10.8<br>p = 0.0238	13.3<br>p = 0.0238	11.6<br>p = 0.0238
ARPA	-	3.0<br>p = 0.0242	13.1<br>p = 0.0121	4.4<br>p = 0.0295	7.6<br>p = 0.0016
RERO	4.3<br>p = 0.0022	27.8<br>p = 0.0238	32.4<br>p = 0.0238	23.4<br>p = 0.0238	27.9<br>p = 0.0238
LIWE	-	21.7<br>p = 0.0121	19.4<br>p = 0.0121	25.6<br>p = 0.0016	26.9<br>p = 0.0016
ICNI	4.6<br>p = 0.0043	22.9<br>p = 0.0238	38.2<br>p = 0.0238	21.1<br>p = 0.0238	20.4<br>p = 0.0238
HV18MK	-	16.2<br>p = 0.0238	26.6<br>p = 0.0238	5.5<br>p = 0.0238	14.0<br>p = 0.0238

Table S6: Cell fraction in G2/M phase: significant change to IR in %.

	Talazoparib + IR		Niraparib + IR	
SBLF7	-		-	
SBLF9	8.6	p = 0.0238	-	
ANST	6.7	p = 0.0238	7.5	p = 0.0238
ARPA	11.9	p = 0.0121	8.4	p = 0.0016
RERO	28.1	p = 0.0238	23.6	p = 0.0238
LIWE	19.3	p =0.0121	26.8	p = 0.0016
ICNI	33.6	p = 0.0357	15.8	p = 0.0357
HV18MK	25.3	p = 0.0238	12.7	p = 0.0238
